# Supplementary material for: Mixed-methods study protocol to develop and pilot a Competency Assessment Tool to support therapists in the care of patients with blunt CHest trauma (CATCh study)
Source: BMJ Open. 2025 Aug 22;15(8):e095438. doi: 10.1136/bmjopen-2024-095438 (PMC12374674; doi:10.1136/bmjopen-2024-095438)
Supplement: Supplementary file 1 [file bmjopen-2024-095438.reviewer_comments.pdf]

PEER REVIEW HISTORY

BMJ Open publishes all reviews undertaken for accepted manuscripts. Reviewers are asked to complete a checklist review form and are provided with free text boxes to elaborate on their assessment. These free text comments are reproduced below.

ARTICLE DETAILS

Title (Provisional)

A mixed methods study protocol to develop and pilot a Competency Assessment Tool to support therapists in the care of patients with blunt CHest trauma (CATCh Study)

Authors

Battle, Ceri; Barnett, Jane; Driscoll, Timothy; Hutchings, Hayley Anne; O’Neill, Claire; Toghill, Hannah; Whelan, Rhys; Baker, E

VERSION 1 - REVIEW

**Reviewer**	**1**
**Name**	**Elbaih, Adel Hamed**
**Affiliation**	**Suez Canal University , associate professor of emergency medicine**
**Date**	**19-Dec-2024**
**COI**	**None**

The manuscript

The data you collected in this manuscript is good. Based on these data, you might have a good study result. Unfortunately, this manuscript does not reach the criteria for publication.

Overall:

1. There are many spelling errors in this manuscript. See guide for authors for a free grammar checker. E.g. in the title CHest trauma (CATCh Study………etc.

2. Every time a new abbreviation is used you should state the full name E.g. CATCh

Title: - A mixed methods study protocol to develop and pilot a Competency Assessment Tool to support therapists in the care of patients with blunt CHest trauma (CATCh Study)

1.Title should be a brief phrase describing the contents of the paper. In addition, please authors should add what the authors are doing in this research and should be no abbreviations.

Please consider providing some additional, specific comments such as:

1. What is the main question addressed by the research?

2. Do you consider the topic original or relevant in the field? Does it address a specific gap in the field?

3. What does it add to the subject area compared with other published material?

4. What specific improvements should the authors consider regarding the methodology? What further controls should be considered?

5. Are the conclusions consistent with the evidence and arguments presented and do they address the main question posed?

6. Are the references appropriate?

7. Please include any additional comments on the tables and figures.

VERSION 1 - AUTHOR RESPONSE

Reviewer: 1

Overall:

1. There are many spelling errors in this manuscript. See guide for authors for a free grammar checker. E.g. in the title CHest trauma (CATCh Study………etc.

> **RESPONSE:** Thank you. We have double checked our manuscript for spelling and grammar.

2. Every time a new abbreviation is used you should state the full name E.g. CATCh

> **RESPONSE:** Thank you. We have ensured that full names are stated prior to the first use of abbreviations

Title: - A mixed methods study protocol to develop and pilot a Competency Assessment Tool to support therapists in the care of patients with blunt CHest trauma (CATCh Study)

1.Title should be a brief phrase describing the contents of the paper. In addition, please authors should add what the authors are doing in this research and should be no abbreviations.

> **RESPONSE:** Thank you. We believe that the title is appropriate for a protocol paper and according to journal requirements.

Please consider providing some additional, specific comments such as:

1. What is the main question addressed by the research?

> **RESPONSE:** Thank you. We are aiming to develop a competency assessment tool that can be used by therapists managing patients with chest injuries. This is already included in the aims and objectives section

2. Do you consider the topic original or relevant in the field? Does it address a specific gap in the field?

> **RESPONSE:** We have already included information in the background section which outlines the specific gap in the field that we are proposing to address. "*Evidence also highlights the importance of healthcare organisations assessing the clinicians' professional competencies. There is a need therefore for valid and reliable assessment tools. Currently, no such tool*

exists for therapists managing patients with blunt chest trauma, and as a result, a lack of standardisation in care exists in the UK".

3. What does it add to the subject area compared with other published material?

RESPONSE: See comment above.

4. What specific improvements should the authors consider regarding the methodology? What further controls should be considered?

RESPONSE: At present, we believe this is the most appropriate methodology for the work we are proposing to undertake. We will however add details to any changes in methodology / protocol in the final papers describing the individual phases of work.

5. Are the conclusions consistent with the evidence and arguments presented and do they address the main question posed?

RESPONSE: We do not have any specific conclusion for this protocol paper, but we will discuss this point in detail in the final papers describing the individual phases of work.

6. Are the references appropriate?

RESPONSE: We believe that we have included the appropriate references for this protocol paper, however we will update each final paper if further evidence emerges during the completion of the studies.

7. Please include any additional comments on the tables and figures.

RESPONSE: We have no further comments to add to the tables and figures
